# Safety of carboxymethylcellulose/polyethylene oxide for the prevention of adhesions in lumbar disc herniation – consecutive case series review

**DOI:** 10.1186/1750-1164-2-2

**Published:** 2008-05-30

**Authors:** Patrick Fransen

**Affiliations:** 1 Department of Neurosurgery, Clinique du Parc Léopold, Bruxelles, Belgium

## Abstract

**Background:**

Epidural fibrosis is regarded as a cause of failed back surgery syndrome (FBSS) when excessive adhesional/fibrotic scar tissue causes compression, pain or discomfort by tethering of nerve tissue to the surrounding muscle or bone. Fibrosis inhibitors could therefore increase the success rate of spinal surgery and decrease the need for reoperations. In recent years, bio-resorbable gels or films for the prevention of peridural fibrosis and post-operative adhesions have been developed that look clinically promising. This included a 100% synthetic, sterile, absorbable gel combinations of carboxymethylcellulose (CMC) and polyethylene oxide (PEO) used to coat the dura to reduce scarring after discectomy which became available in Europe in 2002. However, given the burden of the problem and unfavorable experience with other types of adhesion-reduction agents, our unit decided to evaluate the safety of CMC/PEO in a large population of patients undergoing spinal microdiscectomy for herniation.

**Methods:**

To determine the safety and assess efficacy of carboxymethylcellulose/polyethylene oxide (CMC/PEO) gel as an anti-adhesion gel, a consecutive series of 396 patients undergoing lumbar discectomy performed by one surgeon had CMC/PEO gel administered at the end of surgery. The patients were followed up in accordance with standard clinical practice and records reviewed for side effects, such as skin reactions, general reactions or local fluid collections. Reoperations for recurrent herniation included an evaluation of fibrosis reduction.

**Results:**

No product related complications were observed. Five patients needed reoperations for recurrent herniation. Significant but subjective reduction in fibrosis was observed in these patients.

**Conclusion:**

The findings provide confidence that CMC/PEO gel is well tolerated as an agent to achieve reduction of fibrosis in lumbar disc surgery. Further formal prospective study is recommended in this area of unmet need.

## Background

Although normal healing involves the migration of fibroblasts to a wound site to form a matrix of scar tissue, excessive scar tissue may cause impairment in function, either by direct compression or by tethering of nervous tissues to the surrounding muscle or bone. Such epidural fibrosis is estimated as contributing to 60% of all cases of recurring back pain symptoms in the heterogeneous condition known as failed back surgery syndrome (FBSS) which occurs after discectomy or laminectomy [1]. The direct costs of further diagnostic measures, treatments and repeat surgery, as well as additional societal costs, emphasize the importance of preventive measures during surgery. While spinal surgeons have responded to this challenge by improvements in surgical technique, the problem remains and use of an interposition agent to reduce scarring after discectomy is recognized in the recent Cochrane review as likely to be an important strategy [2].

Recently several types of bioresorbable gels applied directly to the organs and acting as a chemical or physical barrier to surgical adhesions have been approved by licensing regulators. As devices, they are subject to different approval criteria than drug products and since the type of procedures which use these devices are often limited in numbers, the initial safety and efficacy data can often be based on small numbers of patient experiences.

The importance of close monitoring and the collection of safety data when new devices first receive approval for routine use should not be underestimated. Recently, Adcon-L^®^, a promising agent licensed for use as an adhesion-reduction device in laminectomy, was withdrawn after approval due to serious adverse events [3-5] which were not apparent in the early clinical trials [6-8].

In light of such events we decided to prospectively evaluate the safety of a new adhesion-reduction device, carboxymethylcellulose (CMC) and polyethylene oxide (PEO) gel, in a consecutive series of patients undergoing spinal microdiscectomy for herniation. Such a study is in accordance with the essential requirements of the Medical Devices Directive [9] and it is good surgical practice to closely monitor use of any new technique, device or other treatment as it is introduced into routine surgery.

## Methods

CMC and PEO have separately demonstrated anti-adhesion properties [10-12] and have a good safety record in widespread clinical use. The combination CMC/PEO gel is a 100% synthetic gel (OXIPLEX^®^/SP Adhesion Barrier Gel, manufactured by FzioMed, Inc. San Luis Obispo, CA, USA and distributed under the trade names OXIPLEX^®^/SP Adhesion Barrier Gel, DePuy International Ltd, Leeds, UK and MEDISHIELD™ Adhesion Barrier Gel, Medtronic International Trading SARL, Tolochenaz, Switzerland) which received European Class III Device approval and became available for use in Europe in 2002 as an adhesion inhibitor following spinal surgery. Preclinical [13] and initial clinical work [14] with the combination gel showed significant reduction of epidural fibrosis.

In our safety evaluation of this approved agent, a group of 396 patients suffering from one level disc herniation and presenting either with radicular pain resistant to conservative treatment or with radicular pain associated with motor or sensory loss was operated upon by the same surgeon in the same institution (Clinique du Parc Léopold, Brussels, Belgium) between January 1^st ^2003 and December 31^st ^2005. Upon completion of a conventional microdiscectomy, in all patients the decompressed nerve root was systematically covered with a thin layer of [CaCL2 + NaCl + Na carboxymethylcellulose (CMC) + polyethylene oxide (PEO)] gel to prevent excessive growth of scar tissue. Because of previous experience with other anti-adhesion gels, all patients were monitored during the application of the gel looking for changes in blood pressure. They were all reviewed 1 and 6 weeks after surgery. The wound was then subjectively assessed for abnormal healing, redness or subcutaneous collections. Finally, all patients that required reoperations for recurrent disc herniation were carefully evaluated perioperatively for scar tissue formation on the incriminated nerve roots as an assessment of efficacy of the CMC/PEO gel in reducing fibrosis formation. The work was reviewed by the local ethics committee and performed in accordance with the ethical standards laid down in the 1964 Declaration of Helsinki with patients giving their informed consent prior to inclusion in the evaluation.

## Results

Handling and use of the gel was simple. No patient presented with any clinically measurable adverse event during surgery at the time of the application of the gel. The mean length of stay after surgery was 5 days, consistent with normal regional practice. Subjective reviews at both week 1 and week 6 post-surgery demonstrated no local skin reaction and no postoperative fluid collection that required drainage. One patient required reoperation after 13 days for an infection of the wound, but we encountered no other abnormalities in wound healing. Five other patients needed reoperations for recurrent herniation, two after less than a week, one after one month, and two within the first year after surgery.

**Figure 1:**
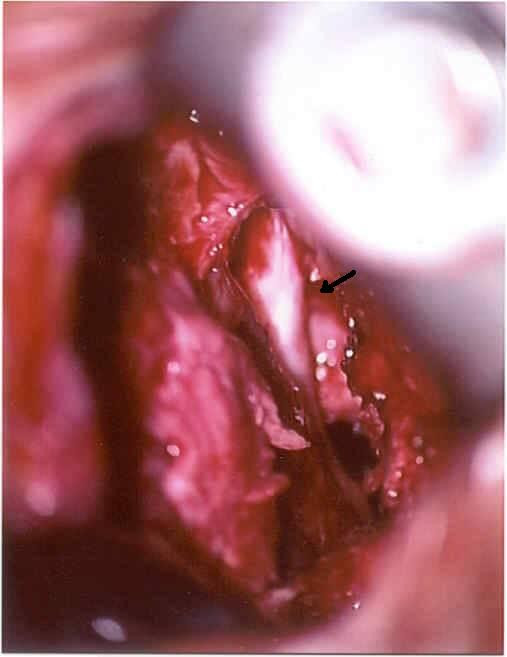
Perioperative view of the operative field for recurrent disc herniation one year after initial surgery with CMC/PEO gel. Note the clear limits of the L5 lumbar nerve root (arrow) and the lack of adherent scar tissue.

In perioperative assessment of the early reoperations, as expected, there was little or no scar tissue. However, although anecdotal, it is worth mentioning that clinically significant fibrosis reduction was observed in the three patients presenting with delayed recurrences – one patient reoperated after one month and the two patients reoperated within the first year. Figure 1 provides a perioperative view of the operative field in the patient who had recurrent disc herniation one year after initial surgery with CMC/PEO gel. Note the clear limits of the L5 lumbar nerve root (arrow) and the lack of adherent scar tissue. This allowed facilitated dissection and separation of the nerve root from the surrounding tissues. At the end of the reoperation CMC/PEO gel was used again. The herniation did not recur and the clinical evolution of this patient was uneventful.

## Discussion

The first gels to be used for prevention of fibrosis were semi-synthetic carbohydrate polymers such as GT 1587 (Adcon-L^®^, Gliatech Inc.). Initial animal models proved favorable [15,16] and subsequent clinical research [6-8] indicated the agent was effective in reducing fibrosis. It is our local policy to closely monitor new agents as they are adopted in routine surgery and with the introduction of GT 1587 we commenced evaluation on a consecutive series of patients in whom we used the agent. After treating 46 patients with GT 1587 we identified three patients with painful erysipeloïd-like skin reactions. Two other patients needed reoperations for unexplained painful subcutaneous fluid collections which proved not to be infection related. The possibility of an exsudative reaction to GT 1587 attributed to its animal gelatin a component was put forward. A local complication rate of 10.8% was found unacceptable in our local risk benefit assessment and we stopped using this gel at an early stage. (Personal presentations; Belgian Neurosurgical Society, 2004). Further reports of serious complications associated with the use of GT 1587 began emerging in the literature after the gel had gone into routine clinical use, including per-operative tachycardia and hypotension [3] and increased rate of CSF leakage [4,5]. GT 1587 was subsequently withdrawn from use.

Our personal experiences with GT1587 highlighted the need for close postoperative vigilance with the use of new products even though it was approved for clinical use as having met the essential requirements of the European Medical Devices Directive [9]. A similar issue recently arose with a promising new anti-adhesion gel in gynecological surgery resulting in withdrawal of the agent after both European and USA approval of the device [17]. On the basis of this latter experience, with the European approval of a subsequent adhesion-reduction solution for gynecological and general surgery, a Europe-wide safety registry was established to allow coordinated close monitoring of the device as it went into routine surgical use [18,19].

The results of our clinical safety assessment of CMC/PEO in spinal surgery were positive. While not a randomized controlled clinical efficacy study, this case series represented responsible safety monitoring of an approved agent. In the case series, we experienced no complications related to use of CMC/PEO gel and only five patients needed reoperations for recurrent herniation, which was lower than our previous experience (Personal presentations Société Francophone de Neurochirurgie du Rachis 2006 and European Association of Neurological Societies 2006). It should be noted that, according to communications with the manufacturer of this device (FzioMed, Inc.), that nearly 100,000 units have been distributed worldwide since the product was introduced in 2002 and thus far, there have been no reports of adverse events that were attributable to the device.

While perioperative bleeding control, avoidance of excessive nerve root retraction and nucleus remnants during surgery, as well as adequate decompression remain the cornerstones of FBSS prevention – we believe the use of an interposition agent is an important element of an overall anti-adhesion strategy.

## Conclusion

In close monitoring of use in a large consecutive case series, there were no adverse events related to the use of the CMC/PEO gel. On the basis of this safety evaluation, we are currently using CMC/PEO on a routine basis for all microdiscectomy procedures as part of our overall anti-adhesion strategy and await the finding of prospective control based efficacy studies with interest.

## Competing interests

No funding was provided for the conduct of this case review. The work was presented as an oral paper at the 2007 American Association of Neurological Surgeons meeting, April 16^th^, 2007. The costs of travel and hotel accommodation were reimbursed by FzioMed, Inc. The article-processing charge from the Annals of Surgical Innovation and Research will be covered by a grant from FzioMed, Inc. who have also provided grant funding to facilitate the support of Corvus Communications in the development of this manuscript.

## Authors' contributions

As sole author I conceived and designed the study evaluation, collected and analyzed the findings, developed the core draft manuscript and have approved the final manuscript.
